# Supplementary material for: Observing Nearby Nuclei on Paramagnetic Trityls and MOFs via DNP and Electron Decoupling
Source: Chemistry. 2022 Oct 11;28(68):e202202556. doi: 10.1002/chem.202202556 (PMC9795816; doi:10.1002/chem.202202556)
Supplement: Supplementary file 1 — Supporting Information [file CHEM-28-0-s001.pdf]

Chemistry–A European Journal

Supporting Information

Observing Nearby Nuclei on Paramagnetic Trityls and MOFs via DNP and Electron Decoupling

Kong Ooi Tan, Luming Yang, Michael Mardini, Choon Boon Cheong, Benoit Driesschaert, Mircea Dincă, and Robert G. Griffin*

Supporting Information

1. 3D-printed adapter for sample preparation

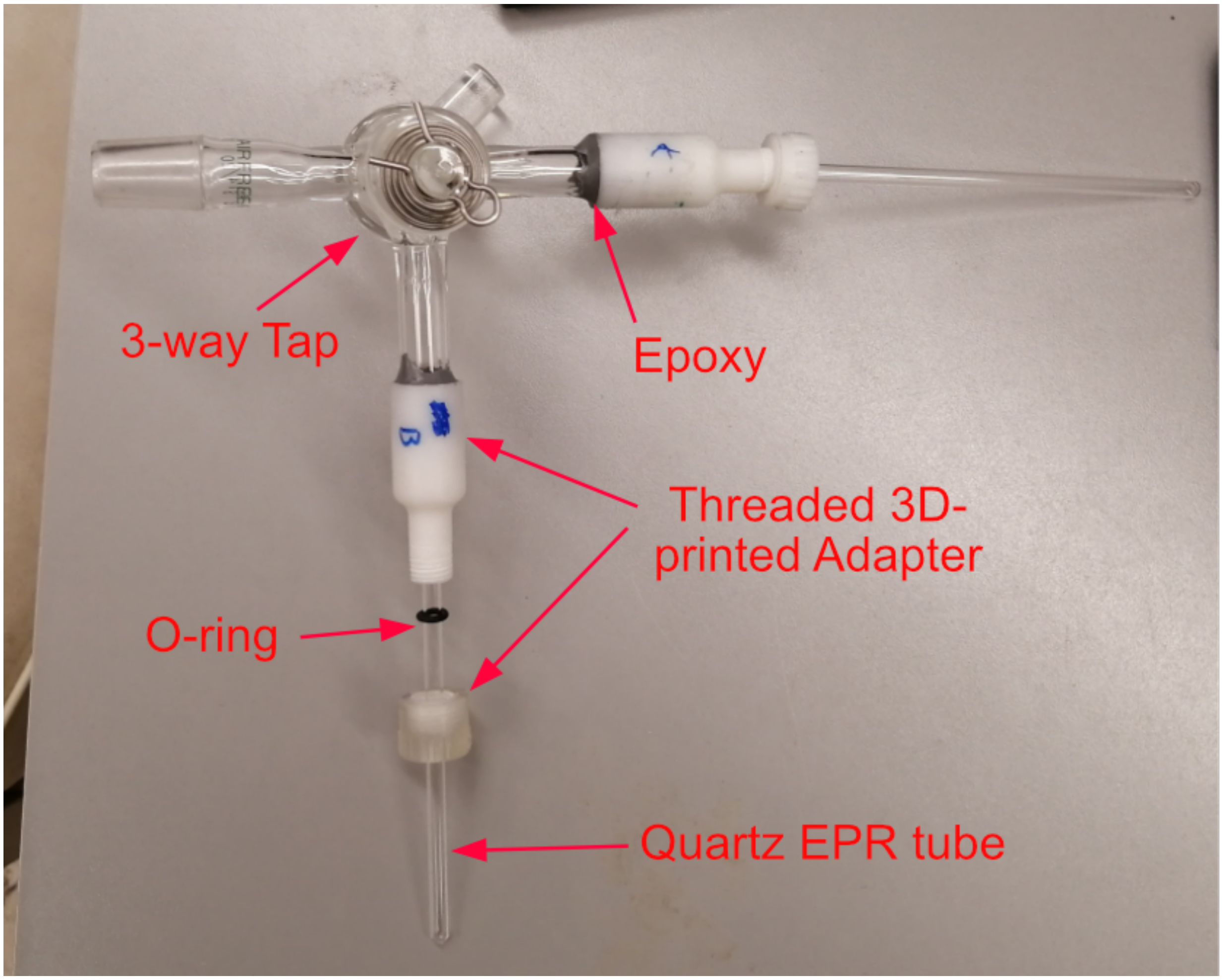

Figure S1. Picture of the homemade 3D-printed adapter printed using either clear or rigid resins with Form 3 (Formlabs Inc.). The two-piece adapter was threaded with a tap and die set to accommodate the O-ring that forms a good seal when the cap is tightened. The setup was tested to be leakproof, i.e., a minimum pressure of 10^{-2} mBar can be sustained while being pumped by a rotary vane pump. The adapter allows two DNP samples to be prepared in a single session.

2. ^{19}F-DNP field profile on ^{2}H-^{19}F Trityls

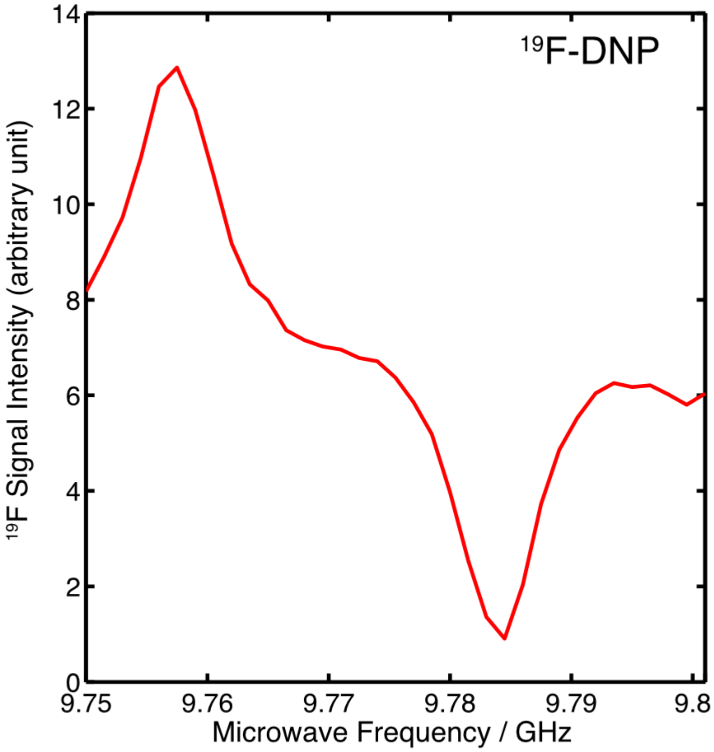

Figure S2. ^{19}F DNP field profile of dFT15F in HFB-trifluoroethanol mixture at 80 K and 0.35 T. As expected, we observed two major peaks separated by $2\omega_{0I}/2\pi$ ~ 28 MHz, which is characteristic for solid effect. The EPR spectrum (data not shown) is similar to OX063 or Finland trityl. We noticed a significant ^{19}F background in the probe (verified by ^{19}F NMR measurement without sample inserted in the probe). Additionally, the solvent is fully fluorinated, which contrasts with only ~10% ^{1}H content in the DNP juice. Thus, we did not determine the ^{19}F DNP enhancement because it is less relevant for electron decoupling [illegible]

3. HRMS spectra of dFT15F

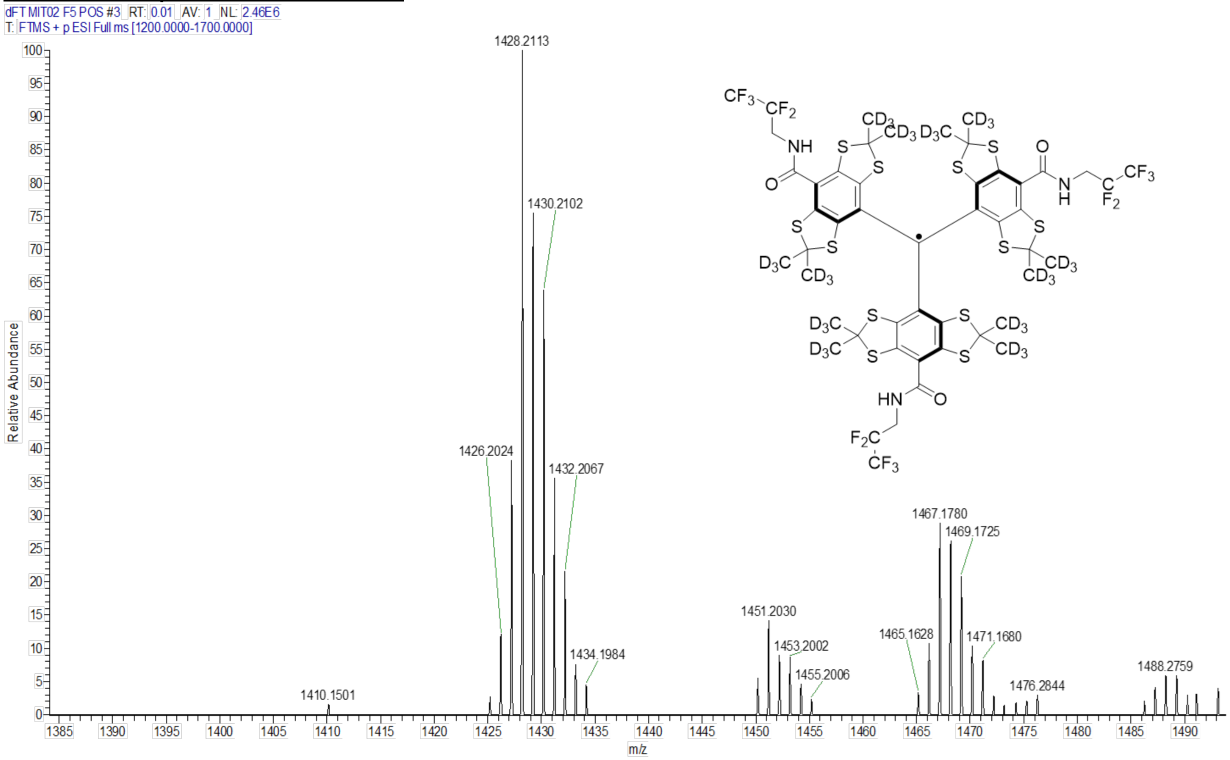

Figure S3. HRMS (ESI pos) spectrum of dFT15F.

4. DFT calculations of FT15F

ORCA was used for DFT calculations as previously described in Tan et. al., (doi:10.1126/sciadv.aax2743). Geometry optimization was done using the B3LYP functional and 6-31G basis set. Calculations of EPR parameters, namely the electron *g*-tensor and hyperfine couplings to all ^{1}H, ^{2}H, and ^{19}F nuclei, were done with the final optimized structure and same functional with the IGLO-III basis set. The e^{-}-^{19}F distances are extracted by first computing the eigenvalues (principal components) of each hyperfine tensor, then subtracting the isotropic component, leaving the diagonal elements of the traceless anisotropic hyperfine interaction that are then used to compute the electron-nuclear distance. We note that some anisotropic hyperfine interactions do not have diagonal elements in the form of [-1, -1, 2] in the principal-axis system, i.e., a point-dipole

approximation is not strictly valid in these cases. The largest deviation observed for e-^{19}F dipolar coupling has diagonal elements of [-0.65, -1.35, 2]. Nevertheless, most e-^{19}F couplings have diagonal elements approximately close to [-1, -1, 2]. We have tabulated the statistics concerning the e-^{19}F of dFT15F in Table S1.

Group	Mean Distance	Standard Deviation	Minimum	Maximum
All ^{19}F atoms	7.6 Å	1.1 Å	6.0 Å	9.1 Å
CF_2	6.8 Å	0.6 Å	6.0 Å	7.7 Å
CF_3	8.2 Å	1.0 Å	6.1 Å	9.1 Å

Table S1 electron-^{19}F distance information determined by DFT calculations.

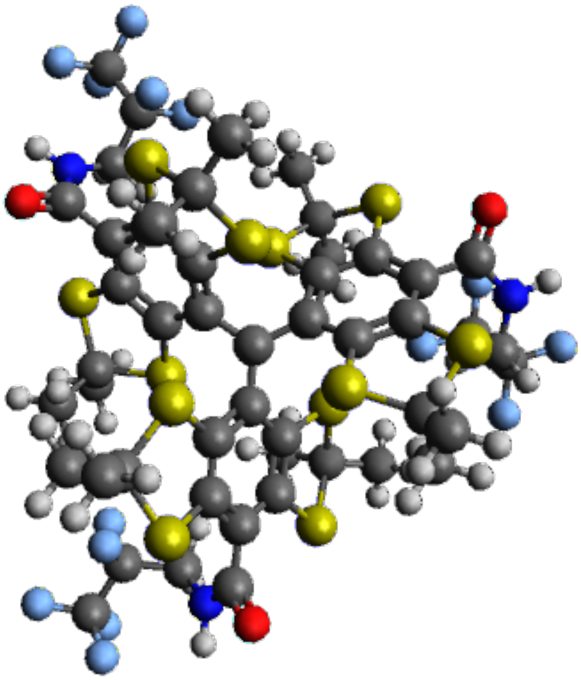

Figure S4. DFT-optimized structure of dFT15F. The atoms are colour-coded as follows: carbon (black), hydrogen (grey), nitrogen (blue), oxygen(red), and fluorine (light blue).
